# Gut Microbiota and the Gut–Liver Axis in Liver Disease: From Chronic Viral Hepatitis to Cirrhosis, Hepatocellular Carcinoma, and Microbiome-Based Therapies

**DOI:** 10.3390/microorganisms13051053

**Published:** 2025-04-30

**Authors:** Sniedze Laivacuma, Olga Oblate, Aleksejs Derovs

**Affiliations:** 1 Faculty of Medicine, Department of Infectology, Rīga Stradiņš University, 16 Dzirciema Str., LV-1007 Riga, Latvia; s.laivacuma@inbox.lv; 2 Riga East University Hospital, 2 Hipokrata Str., LV-1079 Riga, Latvia; 3 Department of Residency, Rīga Stradiņš University, 16 Dzirciema Str., LV-1007 Riga, Latvia; oblateolga@gmail.com; 4 Faculty of Medicine, Department of Internal Diseases, Rīga Stradiņš University, 16 Dzirciema Str., LV-1007 Riga, Latvia

**Keywords:** gut microbiota, gut–liver axis, hepatitis B virus, hepatitis C virus, hepatocellular carcinoma, liver cirrhosis

## Abstract

Chronic viral hepatitis B and C remain major global health challenges, contributing significantly to liver-related morbidity and mortality. Despite antiviral therapies and vaccines for HBV, progression to cirrhosis and hepatocellular carcinoma remains common. For HCV, the lack of a vaccine and high chronicity rates further complicate outcomes. Recent evidence highlights gut–liver axis dysfunction and microbiota dysbiosis in disease progression, immune dysregulation, and fibrosis. Notably, alterations in microbiota composition, including reduced commensal bacteria such as *Bifidobacteria* and *Lactobacilli* and an increase in putatively harmful *Enterobacteriaceae* and *Veillonellaceae*, have been observed in HBV/HCV infections and cirrhosis. While antiviral therapies do not directly target the gut microbiota, they can contribute to partial restoration of microbial balance by reducing hepatic inflammation and improving gut–liver axis integrity. Nonetheless, post-treatment patients remain at elevated risk of HCC due to persistent epigenetic and immune-mediated changes. Emerging interventions, including probiotic strains, prebiotics, and symbiotics, demonstrate potential in enhancing gut health, alleviating inflammation, and enhancing the quality of life for liver disease patients. Moreover, the gut microbiota is gaining increasing recognition as a potential non-invasive biomarker for early disease detection and monitoring. Ultimately, modulating the gut microbiota could become an integral component of future strategies for managing chronic liver diseases and preventing their complications.

## 1. Introduction

In recent years, trials on the gut microbiota have revealed new goals for the prevention and treatment of chronic viral hepatitis. The gut and liver are closely interconnected, communicating through multiple bidirectional pathways within the hepatobiliary system, including the portal vein and systemic circulation. This strong gut–liver relationship forms the basis for the influence of the gut microbiota in regulating liver health. Numerous trials have reported that imbalance in the gut microbiota could play a pivotal role in both triggering and advancing liver diseases. Moreover, gut microbiota diversity has been linked to many liver disorders like autoimmune hepatitis, alcoholic liver disease, chronic hepatitis B, liver cirrhosis, and even hepatocellular carcinoma (HCC). Notably, these microbiota changes vary depending on the specific underlying disease mechanisms [1].

In the meantime, it is crucial to keep in mind the intricate process of bile metabolism and the dynamics of hepatointestinal bile circulation. Bile acids (accounting for 200–600 mg per day) travel from the liver to the gut and are then largely reabsorbed—with approximately 95% returning through enterohepatic circulation. This process plays a pivotal role in different physiological processes like metabolic regulation, nutrient absorption, and overall homeostasis. The remaining 5% is converted into secondary bile acids by the gut microbiota before being transported back to the liver via the portal vein. This intricate interplay between bile metabolism and gut microbiota highlights their mutual influence in maintaining digestive and metabolic balance [2]. This paper aims to present the current state of knowledge on gut microbiota alterations across different stages of HBV and HCV infection.

## 2. Viral Hepatitis and the Gut–Liver Axis

**Chronic viral hepatitis B.**

Chronic viral hepatitis B (HBV) is a well-recognized global health challenge. Even with the availability of vaccines and antiviral therapies, it continues to pose a significant threat to human health worldwide. While some individuals with chronic hepatitis B may remain in an inactive state, the infection can readily progress to severe complications, including cirrhosis, liver failure, hepatocellular carcinoma, and even the need for liver transplantation. Ultimately, it contributes to a high burden of liver-related mortality. As of 2016, only 27 million individuals—representing 10.5% of the global population living with hepatitis B—were aware of their condition. Among those diagnosed, just 4.5 million, or 16.7%, had begun receiving treatment. Chronic hepatitis B can be managed with medications, including oral antiviral agents. While these treatments cannot completely cure the infection, they play a fundamental role in slowing the progression of cirrhosis, reducing the risk of liver cancer, as well as significantly improving long-term survival. Fortunately, only a portion of individuals with chronic hepatitis B—with estimates varying between 10% and 40%, based on the context and the criteria for eligibility—require treatment [3].

The makeup of the gut microbiota plays a role in shaping the host’s immune response to HBV. Consequently, disruptions in microbial structure that lead to immune-related damage appear to be a key factor in the progression to chronic HBV infection and warrant close attention [1].

The progression and persistence of chronic HBV infection are influenced not only by factors such as viral load, pathogenicity, and route of transmission, but also by the host’s immune system and gut microbiota composition. When the host is infected with HBV, the virus is recognized by the relevant toll-like receptors (TLRs), triggering antiviral defense mechanisms. These mechanisms include TLR-mediated recognition, interferon release, activation of natural killer cells, and the production of proinflammatory cytokines. Research into the gut–liver axis has shown that the liver and intestines originate from the same embryonic tissue and are linked anatomically via the portal vein system. Notably, the intestinal tract is believed to play a regulatory role in the progression of chronic HBV infection [4].

Although the liver is without direct interaction with live commensal microbes, continuous exposure to microbe-derived ligands and metabolites through the gut–liver axis plays a pivotal role in shaping liver immunity. Liver sinusoidal endothelial cells recognize lipopolysaccharides (LPS) from Gram-negative bacteria through Toll-like receptor 4, leading to the release of interleukin-10, which promotes T-cell tolerance. Kupffer cells, the most abundant group of tissue-resident macrophages, are also activated via the LPS-TLR4 pathway, triggering the release of immune-suppressing molecules such as IL-10 and transforming growth factor-β, which help regulate immune responses within the liver [5].

The dynamic interplay between commensal microbes and the liver plays a crucial role in shaping hepatic physiology and influencing the body’s response to pathological challenges. Notably, the ability to clear HBV is age-dependent, largely due to the maturation of the gut microbiota, which begins colonizing the gastrointestinal tract immediately after birth.

**Chronic viral hepatitis C.**

Hepatitis C virus (HCV) is a global medical threat, with the highest prevalence reported in the WHO Eastern Mediterranean and European Regions, estimated at 2.3% and 1.5%, respectively, in 2015. HCV can cause both acute and chronic infection, but its ability to remain asymptomatic during the acute phase makes it a persistent public health challenge. Notably, HCV has a markedly higher tendency to progress to chronic infection compared to hepatitis B virus in adult populations, with approximately 70–85% of acute HCV infections becoming chronic, versus 5–10% for adult-acquired HBV. Chronic HCV infection triggers inflammation in hepatocytes, often progressing to fibrosis and increasing the risk of severe liver complications [3,6].

Direct-acting antivirals (DAAs) are now capable of treating the majority of individuals infected with HCV, with treatment courses generally ranging from 8 to 24 weeks, depending on whether cirrhosis is present or absent. One of the significant concerns is still that availability of HCV treatment is improving, but remains too limited. As of 2017, approximately 71 million people worldwide were living with HCV infection. Of these, an estimated 13.1 million (19%) were aware of their condition. Among those diagnosed with chronic HCV, nearly 5 million had received treatment with DAAs by the end of that year [6]. HCV infection induces epigenetic changes that are passed from virus-infected liver cells to their progeny, leading to long-term molecular alterations that can contribute to hepatocellular carcinoma (HCC) development—even after successful viral eradication. Although DAAs have revolutionized the treatment of HCV infection, the incidence of HCV-related HCC is projected to rise in the coming years. While viral elimination helps restore liver function and may reverse mild fibrosis, post-treatment patients remain at an increased risk of developing HCC, necessitating ongoing monitoring and surveillance [7]. Similarly, alterations in the gut microbiota have also been observed in chronic hepatitis C infection, with emerging evidence suggesting a role in disease progression and immune regulation.

## 3. Gut Microbiota Through the Lens of Chronic Viral Hepatitis B and C Progression

The human digestive system hosts a diverse community of microorganisms—including bacteria, protozoa, fungi, archaea, and viruses—collectively known as the gut microbiota. This microbial community represents a remarkably intricate ecosystem, weighing approximately 1–2 kg per person, where trillions of microorganisms coexist in a mutualistic relationship—both with each other and with their human host [8]. Over 90% of the microorganisms in the human gut belong to four dominant phyla: *Bacteroidota* (formerly *Bacteroidetes*), *Firmicutes*, *Proteobacteria*, and *Actinobacteria* [9]. The primary bacterial groups are classified under the genera *Bacteroides*, *Bifidobacterium*, *Ruminococcus*, *Peptostreptococcus*, *Clostridium*, *Faecalibacterium*, *Peptococcus*, and *Eubacterium*. Alongside bacteria, the predominant fungal genera present in the gut include *Saccharomyces*, *Candida*, *Galactomyces*, *Bullera*, *Aspergillus*, *Trametes*, *Pleospora*, *Sclerotinia*, *Penicillium*, and *Rhodotorula* [10,11,12,13].

Throughout an individual’s life, the configuration of the gut microbiota undergoes continuous transformation, influenced by different factors—including the mode of delivery at birth, breastfeeding, dietary shifts, and frequent or prolonged use of antibiotics and other medications. This dynamic microbial community plays a crucial role in maintaining overall homeostasis, facilitating nutrient and energy extraction from food, and generating essential metabolites that support the host’s metabolic functions [14,15].

Published research on how viral hepatitis B and C infections affect the gut microbiota remains limited. Additionally, the role of microbial imbalance in the progression of the disease is still not completely clear.

Chronic liver inflammation induced by HBV and HCV—though these viruses are strictly hepatotropic and employ immune evasion strategies—can secondarily impact gut homeostasis. This occurs through systemic inflammation, altered bile acid signaling, and gut–liver axis disruption, ultimately contributing to gut dysbiosis [16]. In the context of liver cirrhosis, compromised liver perfusion and functional decline compromise detoxification capacity, facilitating intestinal bacterial overgrowth and systemic inflammation. This exacerbates the intricate system of bidirectional interactions between host immunity and the gut microbiota. Moreover, both HBV and HCV infections promote oxidative stress within hepatocytes, causing the accumulation of reactive oxygen species and cytotoxic metabolites, which contribute to cellular damage in both hepatic and intestinal epithelial tissues [17,18].

In a trial performed by Perez-Matute et al., it was noted that among individuals affected by chronic hepatitis C virus infection and lower grades of fibrosis (F0-1), use of direct antiviral drugs (DAAs) could restore previous intestinal bacterial changes [19]. For example, Entecavir administration contributes to an increased representation of *Clostridium sensu stricto* 1, a genus associated with higher levels of high-density lipoproteins in the large and extra-large subclasses and a reduced threat of cardiovascular disease. Hepatic lipid accumulation and steatosis may contribute to the onset of inflammation, which, if sustained, can progress to cirrhosis and concurrently elevate oxidative stress within hepatic tissues [20,21]. The findings emphasize a significant interconnection between the liver and the intestine, suggesting that slight disruptions in the intestinal microbiome caused by liver injury can be effectively counteracted through the use of appropriate drugs [14].

Administration of direct-acting antivirals in individuals with liver cirrhosis appears to exert beneficial effects on intestinal microbiota composition, along with hepatic fibrosis and inflammation; however, the data exhibit variability and still lack definitive trends. DAA therapy markedly reduces the proportion of *Enterobacteriaceae*, *Staphylococcus*, and *Veillonellaceae*. Notably, the naturally lower density of *Enterobacteriaceae* in the healthy distal gut primarily reflects the hypoxic environment, which favors the growth of obligate anaerobic bacteria such as *Clostridia* and *Bacteroidia*. In addition, *Enterobacteriaceae* lack the enzymatic capacity to degrade complex carbohydrates efficiently, relying instead on the passive diffusion of oligosaccharide molecules. Together, these factors contribute to their limited abundance under healthy conditions. *Veillonellaceae* represents a key microbial taxon correlated with fibrosis severity, particularly in non-obese individuals. Members of this family are capable of producing propionate, a major short-chain fatty acid (SCFA), which is thought to play a role in the development of chronic liver disease. Moreover, LPS and SCFAs derived from intestinal *Veillonella* promote the production of proinflammatory cytokines, including IL-6 and IL-10, alongside TNF-α, in peripheral blood mononuclear cells derived from humans, thereby contributing to liver-specific inflammation and disease progression [14,22,23,24].

**Chronic viral hepatitis without cirrhosis.**

The gut microbiota plays a crucial role in maintaining human physiological balance, and its disruption has been increasingly associated with the development of various diseases, including chronic liver conditions. Alterations in the composition of intestinal flora have been observed in individuals carrying chronic HBV, patients with active chronic hepatitis B, and those with HBV-related liver cirrhosis. The structures and abundances of the bacterial groups were notably different. Individuals with chronic hepatitis B and cirrhosis, especially, show a marked reduction in *Bifidobacteria* and *Lactobacillus* populations, while levels of *Enterococcus* and *Enterobacteriaceae* are significantly elevated compared to healthy controls [25].

The advancement of liver diseases, especially liver cirrhosis, is strongly influenced by bacterial metabolites originating from the gut. Furthermore, the gut microbiota plays an essential role in shaping systemic immune responses. A study conducted by researchers in China demonstrated that sterilization of the gut microbiota using antibiotics impaired the ability of adult mice to clear HBV infection. In contrast, adult mice with intact gut microbiota successfully cleared HBV within six weeks post-infection. These findings suggest that the gut microbiota play an essential role in facilitating immune responses against HBV [5]. In another study where the gut microbes of chronic hepatis B patients and healthy individuals were studied, data revealed that there was a notable reduction in *Bacteroides* populations in hepatitis patients compared to healthy individuals. Furthermore, the authors concluded that the configuration of the intestinal microbiota in patients with chronic hepatitis B undergoes significant alterations compared to its state before severe liver injury. These compositional changes in the gut microbiota suggest a putative role in pathogenesis in the progression of chronic HBV infection [26]. Additionally, another study showed that the genus *Megasphaera*, a member of the phylum *Firmicutes* and *Anaerostipes*, as butyrate-producing bacteria, was relatively abundant in chronic viral hepatitis among individuals with high ALT levels relative to those with normal ALT [27].

Alterations in the microbiome were also evident in HCV patients, potentially driven by bacterial translocation [28]. Even mild HCV infection has been associated with alterations in the gut microbiota. In cases without evidence of liver cirrhosis, increased levels of *Bacteroides* and *Enterobacteriaceae* were observed. The same study also reported a reduction in *Clostridiales* and an elevated abundance of *Lactobacillus* and *Streptococcus* (specifically viridans group *streptococci*) in individuals with early-stage HCV infection [29]. HCV-infected patients had less abundance of *Firmicutes*, *Proteobacteria*, and *Actinobacteria* than healthy controls [30].

A recent trial demonstrated that patients with liver fibrosis are likely to exhibit an imbalance in the gut microbiota, characterized by increased numbers of *Staphylococcus*, *Streptococcus*, *Enterococcus*, *Phascolarctobacterium*, *Escherichia*, *Proteus*, and *Ralstonia*, and decreased numbers of *Eubacterium*, *Faecalibacterium*, *Dorea*, *Bifidobacterium*, *Bacteroides*, *Alistipes*, *Barnesiella*, and *Prevotella* [31].

**Directly acting antivirals and microbial shifts.**

With the growing clinical use of direct-acting antivirals for HCV and nucleos(t)ide analogs for HBV, the impact of these antiviral treatments on the gut microbiota has attracted increasing attention in recent research.

A prospective cohort study conducted by Hsu et al. in Taiwan investigated gut microbiota composition before and after DAA therapy in HCV-infected individuals [32]. The study enrolled 126 participants, comprising 42 HCV-infected patients and 84 uninfected controls. Prior to viral eradication, the gut microbiota of chronically HCV-infected individuals—regardless of cirrhosis status—differed significantly from uninfected controls. Notably, after 12 weeks of DAA therapy, microbial shifts were observed, including increased relative abundances of *Coriobacteriaceae*, *Staphylococcaceae*, *Peptostreptococcaceae*, and *Succinivibrionaceae*, and a reduction in *Morganellaceae*, *Pasteurellaceae*, and *Moraxellaceae*. However, these changes were modest, suggesting that DAA treatment alone exerts a limited impact on restoring the gut microbiota toward a healthy profile [32].

Similarly, a study by Pérez-Matute et al. reported that DAA therapy did not significantly alter the overall gut microbiota composition in 22 non-cirrhotic HCV-infected patients [19].

More recently, Huang et al. evaluated the intestinal microbiota in 120 HCV-infected patients, the majority without significant fibrosis or cirrhosis. Interestingly, prior to DAA treatment, patients exhibited a higher relative abundance of taxa such as *Ruminococcaceae*, *Eubacterium*, *Agathobacter*, *Alistipes*, *Bifidobacterium*, and *Klebsiella* compared to controls—findings that differ from earlier studies [19,32]. These discrepancies likely reflect inter-study variability related to host genetics, diet, immune status, fibrosis stage, and environmental exposures. Importantly, Huang et al. concluded that the gut microbiota profile did not undergo significant changes even six months after successful viral eradication, emphasizing the need for longer-term follow-up studies [33].

Additionally, in patients co-infected with HCV and HIV, nuanced shifts in gut microbiota composition were observed after antiviral therapy. Increases in beneficial genera such as *Subdoligranulum*, *Lachnospira*, and *Phascolarctobacterium* were noted, particularly among responders with low-grade hepatic fibrosis. However, these improvements were absent in nonresponders, suggesting that hepatic fibrosis status may be a critical determinant of microbiota recovery following treatment [34].

Collectively, these findings indicate that while DAA therapy achieves robust viral clearance, its effects on gut microbiota restoration are likely modest and heavily influenced by the presence or absence of advanced liver fibrosis rather than by DAA therapy per se (see Table 1).

Research performed by Ponciani et al. showed a marked improvement in gut microbiota dysbiosis following successful eradication of HCV in cirrhotic patients treated with DAAs. This improvement was largely attributed to the restoration of hepatic function post-viral clearance. The observed changes included increased microbial diversity, aligning more closely with that of healthy individuals, a reduction in HCV-associated potentially pathogenic bacterial taxa, and decreased circulating levels of proinflammatory cytokines and chemokines. Specifically, viral eradication was associated with reduced levels of potentially harmful bacterial genera, including *Enterobacteriaceae*, *Staphylococcus*, and *Veillonellaceae* [22]. For further details, please see Table 1.

## 4. Virus-Related Liver Cirrhosis and Microbiota Composition

Cirrhosis is linked to a compromised immune response, which may contribute to dysbiosis or shifts in the microbiota detected in various sample types such as stool, mucosal tissue, ascitic fluid, liver, blood serum, and saliva [36]. There is a notable increase in the relative abundance of bacterial families such as *Enterobacteriaceae* (which comprises gram-negative rods like *Escherichia coli* and *Klebsiella*), *Enterococcaceae* (including *Enterococcus faecalis* and *E. faecium*), and *Streptococcaceae*, accompanied by a reduction in potentially beneficial groups like *Lachnospiraceae* and *Ruminococcaceae* [37]. In other studies by the same group, we can see that the recto-sigmoid mucosal microbiota in patients with liver cirrhosis exhibits a significantly decreased presence of indigenous commensal bacteria, specifically *Subdoligranulum*, *Dorea*, and members of *Incertae Sedis* XIV, while showing an elevated abundance of opportunistic or potentially pathogenic genera such as *Enterococcus*, *Clostridium*, *Burkholderia*, and *Proteus* [38]. A separate trial reported elevated levels of specific bacterial genera—including *Veillonella*, *Megasphaera*, *Dialister*, *Atopobium*, and *Prevotella*—in the gut microbiota of individuals diagnosed with liver cirrhosis [39].

There are data available from authors from China who proposed to analyze the intestinal *Bifidobacterium* profile in individuals with chronic liver disease caused by hepatitis B virus; the authors concluded that *Bifidobacterium dentium* seemed to be specifically enhanced, but *Bifidobacterium catenulatum/Bifidobacterium pseudocatenulatum* exhibited a lower rate of occurrence in patients with HBV-related cirrhosis compared to the controls [40].

Beyond alterations in microbial composition, cirrhosis is also associated with functional changes in the gut microbiota. These include increased endotoxin production, impaired conversion of primary to secondary bile acids, and reduced synthesis of short-chain fatty acids. Collectively, these functional disruptions may have clinically significant consequences, potentially contributing to systemic inflammation, immune dysregulation, and further progression of liver disease [41].

## 5. Gut Microbiome and Hepatocellular Carcinoma

Hepatocellular carcinoma (HCC) typically develops in the final stages of liver disease, with the exception of chronic hepatitis B, where it can arise even in the absence of cirrhosis. HCC has been associated with a wide range of etiological risk factors and cofactors, with cirrhosis preceding HCC development in approximately 80–90% of cases [42]. With a global mortality rate of 9.1%, HCC ranks as the fifth most common cancer and represents a significant global health burden. Chronic viral hepatitis—HBV and HCV—is recognized as the primary driver of HCC pathophysiological progression [43]. Patients with HCV-related cirrhosis face a notable risk of progressing to hepatocellular carcinoma, with the disease emerging in a considerable proportion annually. Importantly, even in the absence of cirrhosis, long-term chronic HCV infection can still lead to HCC development in a meaningful subset of individuals over time, underscoring the oncogenic potential of the virus itself [44]. For HBV, in the absence of treatment, an estimated 20% of chronically infected patients remain susceptible to advancing toward cirrhosis [45]. Among patients with HBV infection, the annual incidence of HCC remains below 1% in the absence of cirrhosis, but rises to approximately 2–3% in those with established cirrhotic disease [46].

Overall, the intestinal microbiome in HCC cases is significantly altered. For example, mouse models have shown that mice with HCC have higher populations of *Clostridium* bacteria, as well as *Escherichia coli* overgrowth. Mouse models also indicate a possible migration of *Vietcobacter* species into HCC tissues. In addition, the class *Clostridia*, particularly *Clostridium cluster XIVa*, along with the phylum *Proteobacteria*, showed a significant positive correlation (i.e., increased abundance) in association with HCC. Data are available describing the relationship between the use of broad-spectrum antibiotics and the reduction in the progression of hepatitis HCC in mice [47,48].

In HCC cases related to HBV, a significant reduction in *Proteobacteria*, *Prevotella*, *Faecalibacterium*, *Pseudobutyrivibrio*, *Lachnoclostridium*, and *Ruminoclostridium* has been observed, accompanied by increased levels of Escherichia, Shigella, and Enterococcus. In contrast, in patients with HCV-related cirrhosis—a known precursor state to HCC—the gut microbiota is characterized by a decreased abundance of *Bacteroidota* (formerly *Bacteroidetes*) and elevated levels of *Prevotella*, *Enterococcus*, *Staphylococcus*, *Veillonella*, *Proteobacteria*, *Megasphaera*, *Burkholderia*, and *Fusobacteria*. These microbial shifts in cirrhotic patients may contribute to the pro-inflammatory milieu that promotes hepatocarcinogenesis [18,49].

In a study by Jinato T. et al., researchers compared the gut microbiota composition between patients with viral-related HCC and those with non-hepatitis B or C-related HCC. The microbial profile comprised 11 genera, notably *Faecalibacterium*, *Agathobacter*, and *Coprococcus*, which were identified in the viral-related HCC group. In contrast, five genera, including *Bacteroides*, *Streptococcus*, the *Ruminococcus gnavus* group, *Parabacteroides*, and *Erysipelatoclostridium*, were abundant in non-viral HCC. In patients with HCC unrelated to viral hepatitis, gut microbiota profiles are characterized by a reduced abundance of various short-chain fatty acid-producing bacteria, lower fecal butyrate concentrations, and elevated plasma surrogate markers of microbial translocation. The results of this study align with several earlier investigations on HCC cases, particularly those related to NAFLD etiology [50,51,52,53]. The obtained data might be of potential use in examining intestinal microbiota compositions for diagnosis and treatment modalities in different HCC subgroups.

In a study by He Y et al., the microenvironment associated with HCC was analyzed, and the presence of bacteria within immune cells was verified. Analysis revealed 11 bacterial genera with significantly altered abundance between tumoral and adjacent non-tumoral (paracancerous) tissues, of which four were found to be significantly enriched in hepatocellular carcinoma samples [54]. In addition, there are data about *Ruminococcus gnavus* presence in viral etiology HCC that indirectly promotes TNF-α production, which is a known factor of HCC carcinogenesis [55].

Recent insights emphasize that gut microbiota dysbiosis plays a central role in the development and progression of hepatocellular carcinoma (HCC). Alterations typically include a reduction of beneficial short-chain fatty acid-producing bacteria and an expansion of pathogenic taxa associated with inflammation and immune dysfunction. These microbial shifts contribute to gut barrier disruption, enhanced microbial translocation, and the creation of a pro-carcinogenic hepatic environment. Modulating the gut microbiota through approaches such as probiotics, prebiotics, or fecal microbiota transplantation represents a promising therapeutic strategy under active investigation. Targeting the gut–liver axis may offer new opportunities for preventing and managing HCC in the future [56].

## 6. Changing Microbiome as Treatment for Liver Disease

Gut microbiota dysbiosis in cirrhosis has been implicated as a key factor in driving disease progression, potentially influencing the shift from stable outpatient management to hospitalization. The gut microbiota is shaped by a variety of factors, including dietary patterns, ethnicity, disease stage, and medication use. Among cirrhotic outpatients, greater gut microbiota diversity has been associated with a Middle Eastern dietary pattern, particularly one that includes daily intake of fermented milk products, in contrast to the reduced diversity observed in Western diets. Moreover, higher intake of coffee, tea, and vegetables correlated with a lower hospitalization rate and increased gut microbial diversity, as observed in comparative analyses between Turkish and American patient cohorts [57]. A key contributor to gut dysbiosis—characterized by the presence of bacteria typically found in the oral cavity, such as *Streptococcaceae*, *Veillonellaceae*, and *Porphyromonadaceae*, in stool samples—is the use of proton pump inhibitors (PPIs) [58]. Therefore, the following steps could be recommended: (1) Withdraw redundant PPI use; (2) promote the inclusion of probiotic strains through foods aligned with a Mediterranean dietary pattern; (3) avoid unnecessary therapies, including unregulated or over-the-counter probiotic supplements; and (4) emphasize the importance of dental hygiene and encourage regular oral health care in patients.

One significant complication of liver cirrhosis is hepatic encephalopathy, which substantially reduces quality of life and life expectancy. The use of a probiotic combination consisting of *Clostridium butyricum* and *Bifidobacterium infantis* has been evaluated in patients with HBV-related liver cirrhosis for its effectiveness in treating minimal hepatic encephalopathy. The results indicate that the probiotic may strengthen the intestinal barrier, contribute to ammonia reduction, and support cognitive function improvement [14,59]. Further details are provided in Table 2.

Another possible way to help patients with liver cirrhosis may be prebiotics.

Originally identified as indigestible dietary components, prebiotics are recognized for their ability to enhance host health through the selective stimulation of beneficial microbial populations within the gastrointestinal tract. Specifically, prebiotics are food-derived substances that promote the proliferation and metabolic activity of health-promoting gut microbes, thereby supporting gastrointestinal and systemic health. By nourishing the gut microbiota, these dietary ingredients lead to the production of metabolites such as short-chain fatty acids, which enter systemic circulation and influence not only intestinal health, but also the function of various extraintestinal organs. However, most of the current studies are performed in animal models. One of the prebiotics—garlic polysaccharides—used in an animal model had an impact on the gut microbiota, associated with a growth of *Lachnospiraceae* and *Lactobacillus* alongside a reduction in *Facklamia* and *Firmicutes* [67]. Another prebiotical compound—polysaccharides from *Grifola frondosa*—decreased various blood markers such as ALT, AST, MDA, and TBIL, as well as immune modulators TNF-α, IL-1β, and IL-6, and elevated Superoxide dismutase and Glutathione [68]. In another study, olive oil combined with *Lycium barbarum* polysaccharide showed a decrease of TGF-β1, TNF-α, and Timp-1 and, in turn, an increase of IL-10 and IL-10/TNF-α [68].

Synbiotics, defined as the synergistic combination of probiotic strains and prebiotics, were developed to address the potential survival challenges faced by probiotic strains, aiming to enhance their colonization and beneficial effects within the host gut environment. In a study relevant to the topic, cirrhosis patients with minimal hepatic encephalopathy, but not overt hepatic encephalopathy, were treated with synbiotics, which consisted of a mixture of four probiotic strains (*P. pentoseceus* + *L. mesenteroides* + *L. paracasei* + *L. plantarum*) alongside a combination of three fibers—β-glucan, pectin, and resistant starch—was linked to a decrease in serum total bilirubin levels and an increase in serum albumin, indicating potential improvements in liver function [69].

Owing to its non-invasive characteristics, the microbiome is increasingly being investigated as a candidate biomarker for early detection of chronic liver disease in individuals at risk. Its diagnostic utility may enhance early detection strategies, support risk stratification, and contribute to improved disease monitoring and clinical outcomes [18].

## 7. Conclusions

The gut–liver axis is critically involved in the pathogenesis of liver diseases, contributing to disease development and progression across the entire spectrum, from initial fibrotic changes to end-stage cirrhosis. Liver disease has been increasingly linked to the gut microbiome, and the potential for therapeutic modulation of the microbiome is anticipated to significantly influence future advancements in disease management and overall health outcomes in the medical field. Thus, assessing the impact of gut microbiota modulation represents a critical step in advancing liver disease management. However, at present, studies on gut microbiota are rather heterogeneous, and many of them are performed in vitro or using animal models.

Advancing clinical outcomes in liver disease requires a thorough understanding of its underlying pathology, particularly given the central role of integrated signaling pathways in the progression of fibrosis and cirrhosis. Continued research using clinically relevant models is essential to deepen our understanding of gut microbiota–host immune system interactions, which may uncover key mechanisms in the pathogenesis of liver fibrosis and inform the development of innovative therapies targeting immune responses or the gut microbiome. To better assess the efficacy and mechanistic basis of probiotic strains, prebiotics, and synbiotics, upcoming clinical trials should integrate metagenomic and metabolomic approaches, enabling a deeper understanding of their impact on host physiology and microbial dynamics.

## Figures and Tables

**Table 1:** Effects of antiviral therapy on gut microbiota profiles in chronic hepatitis C and B infections.

**A. Direct-Acting Antivirals (DAAs) for HCV Infection**			
Clinical Context	Increase	Decrease	Ref.
HCV without cirrhosis (DAA therapy)	*Phylum Firmicutes*, genera *Lachnospira*	-	Hsu et al. [32]
HCV with cirrhosis (DAA therapy)	–	*Enterobacteriaceae*, *Staphylococcus*, *Veillonellaceae*	Pérez-Matute et al. [19]
HCV patients post-DAA therapy (majority without cirrhosis)	No significant microbiota change; slight trends in *Ruminococcaceae*, *Eubacterium*, *Agathobacter*, *Alistipes*, *Bifidobacterium*, *Klebsiella*	–	Huang et al. [33]
HCV with/without HIV co-infection, post-DAA therapy	Increased *Subdoligranulum*, *Lachnospira*, *Phascolarctobacterium* (among low-grade fibrosis responders)	No restoration among nonresponders	Chuaypen N et al. [34]
**B. Entecavir Therapy for HBV Infection**			
Clinical Context	Increase	Decrease	
Chronic HBV infection under entecavir therapy	*Clostridium sensu stricto 1*, *Erysipelotrichaceae UCG-007*, *Intestinibacter*	*Streptococcus*, *Atopobium*, and *Murdochiella*	Yu-Xia Lu et al. [35]

Table 1 summarizes the reported changes in gut microbiota composition associated with antiviral therapies in chronic viral hepatitis. Due to the distinct nature of hepatitis B virus (HBV) and hepatitis C virus (HCV) infections, effects of direct-acting antivirals (DAAs) are listed separately from nucleos(t)ide analog therapy for HBV (e.g., entecavir). DAA regimens varied among studies. Specific antiviral agents used (e.g., sofosbuvir-based, glecaprevir-based therapies) are detailed in the main text where available.

**Table 2:** Summary of key clinical studies investigating probiotic strains interventions in liver diseases.

Disease/Condition	Probiotic Strains	Duration	Observed Effects	Reference	Limitations
**Cirrhosis with Hepatic Encephalopathy**	VSL#3—mixture of probiotic strains (*Lactobacillus acidophilus*, *L. delbrueckii* subsp. *bulgaricus*, *L. casei*, *L. plantarum*, *Bifidobacterium breve*, *B. longum*, *B. infantis*, *Streptococcus salivarius* subsp. *thermophilus*)	6 months	Reduced hospitalization rates; improved Child–Pugh and MELD scores; decreased inflammatory markers (IL-1β, IL-6, TNF-α); lower levels of indole, renin, aldosterone, and brain-type natriuretic peptide; trend toward fewer overt hepatic encephalopathy episodes	Dhiman RK et al. [60]	Single-center study; moderate sample size; potential for selection bias
**Cirrhosis without Overt Hepatic Encephalopathy**	Commercial probiotic mixture including *Lactobacillus* and *Bifidobacterium* species	3 months	Improved Child–Pugh score; reduced incidence of overt hepatic encephalopathy; decreased small intestinal bacterial overgrowth, orocecal transit time, and arterial ammonia levels	Pereg D et al. [61]	Limited strain specificity; short duration; lack of long-term follow-up
**Minimal Hepatic Encephalopathy**	*Lactobacillus rhamnosus* GG	8 weeks	Decreased *Enterobacteriaceae*; reduced endotoxemia and TNF-α levels; increased abundance of *Clostridiales* and *Lachnospiraceae*	Bajaj JS et al. [62]	Small sample size; short intervention period; need for larger multicenter trials
**HBV-Related Cirrhosis with Minimal Hepatic Encephalopathy**	*Bifidobacterium longum*, *Lactobacillus acidophilus*, *Enterococcus faecalis*	3 months	Reduced *Enterococcus* and *Enterobacteriaceae*; decreased ammonia levels; improved cognitive function and quality of life; increased abundance of *Clostridium cluster I* and *Bifidobacterium*	Xia X et al. [59]	Limited to HBV-related cirrhosis; small cohort; regional dietary factors may influence results
**Cirrhosis at risk of hepatic encephalopathy**	Commercial probiotic mix (*Lactobacillus*, *Bifidobacterium*, *Streptococcus* spp.)	6 months	Significant reduction in the development of hepatic encephalopathy; improved cognitive function; improved Child–Pugh score	Lunia MK et al. [63]	Single-center study; commercial probiotic composition not fully detailed
**HBV-related cirrhosis for HCC prevention**	Various probiotics (specific strains not detailed)	Median follow-up approximately 5 years	Probiotic therapy associated with a significantly reduced risk of hepatocellular carcinoma (HCC)	Shi K et al. [64]	Retrospective study design; probiotic strains not specified; possible residual confounding
**Minimal hepatic encephalopathy in cirrhosis**	Commercial probiotic mix (including *Lactobacillus acidophilus*, *Bifidobacterium bifidum*)	3 months	Improvement in minimal hepatic encephalopathy; comparable efficacy to lactulose; improvement in cognitive scores	Sharma P et al. [65]	Open-label design; commercial probiotic preparation; limited strain specification
**Liver disease patients undergoing liver resection**	Commercial probiotic mixture (strains not fully detailed)	1 week preoperatively	Reduced postoperative infections; no major difference in inflammatory markers or endotoxin levels	Roussel E et al. [66]	Small sample size; strains not clearly specified; short preoperative duration

Data summarized from published clinical trials as referenced.

## Abbreviations

The following abbreviations are used in this manuscript.

HCC	Hepatocellular carcinoma
HBV	Hepatitis B Virus
TLR	Toll-like receptors
LPS	Lipopolysaccharides
HCV	Hepatitis C Virus
DAA	Directly acting antivirals
SCFA	Short-chain fatty acid
PPI	Proton pump inhibitors
HIV	Human immunodeficiency virus
